# Sebaceous Carcinoma as a Presentation of Muir-Torre Syndrome

**DOI:** 10.7759/cureus.77386

**Published:** 2025-01-13

**Authors:** Emily Saurborn, Bukola Adeshina, Isabella G Stuart, Shane Cook

**Affiliations:** 1 Department of Dermatology, Marshall University Joan C. Edwards School of Medicine, Huntington, USA

**Keywords:** adenoma, carcinoma, epithelioma, germline, immunohistochemistry, microsatellite instability, mismatch repair, muir-torre syndrome, sebaceous neoplasms

## Abstract

Muir-Torre syndrome (MTS) is a rare, autosomal dominant condition that is within the spectrum of Lynch syndrome (hereditary nonpolyposis colorectal cancer (HNPCC)). Sebaceous adenomas are among the most specific manifestations of MTS. Other malignancies include tumors of the colon, rectum, and genitourinary systems, such as endometrial, ovarian, urothelial, and prostate cancer. Individuals at risk for MTS are identified using the Mayo score, which assesses risk based on family history of Lynch syndrome-associated cancers, personal history of these cancers, and age at diagnosis of a sebaceous adenoma or visceral malignancy. We present a case of a firm, red-yellow papule on the upper extremity, which was revealed by a biopsy to be a sebaceous adenocarcinoma. Immunohistochemistry was significant for the loss of MSH2 and MLH1.

## Introduction

Muir-Torre syndrome (MTS) is a rare autosomal dominant genetic variant of hereditary non-polyposis colorectal cancer, or Lynch syndrome, with both visceral and cutaneous involvement [1]. The syndrome was first described by Muir in 1967 and again by Torre in 1968 [1]. Occurrence is due to mutations in DNA mismatch repair (MMR) genes, MLH1, MSH2, MSH6, and PMS2, resulting in microsatellite instability [1,2]. Hallmark dermatological manifestations of MTS include sebaceous adenomas, sebaceous epitheliomas, sebaceous carcinomas, and keratoacanthomas that may or may not include sebaceous differentiation, typically presenting on the head and neck [1,2]. Often, benign cutaneous lesions are the initial presentation of MTS [3]. On physical exam, sebaceous adenomas and carcinomas present as yellowish or skin-colored papules. Visceral malignancies most commonly include colorectal cancers but may also be comprised of cancers of the endometrium, ovaries, cervix, breast, uroepithelium, brain, blood, lung, small bowel, pancreas, hepatobiliary tract, and gastric organs [1,2,4].

Early detection of the various presentations of the MTS is important for the workup of other visceral malignancies to ensure that patients receive integrative care across a multitude of specialties. Thus, we present a case of a patient with a small papule with a biopsy consistent with sebaceous adenocarcinoma. This case underscores the importance of providers being aware of presentations of various syndromes in order to ensure appropriate workup and timely referrals for patients.

## Case presentation

**Figure 1:**
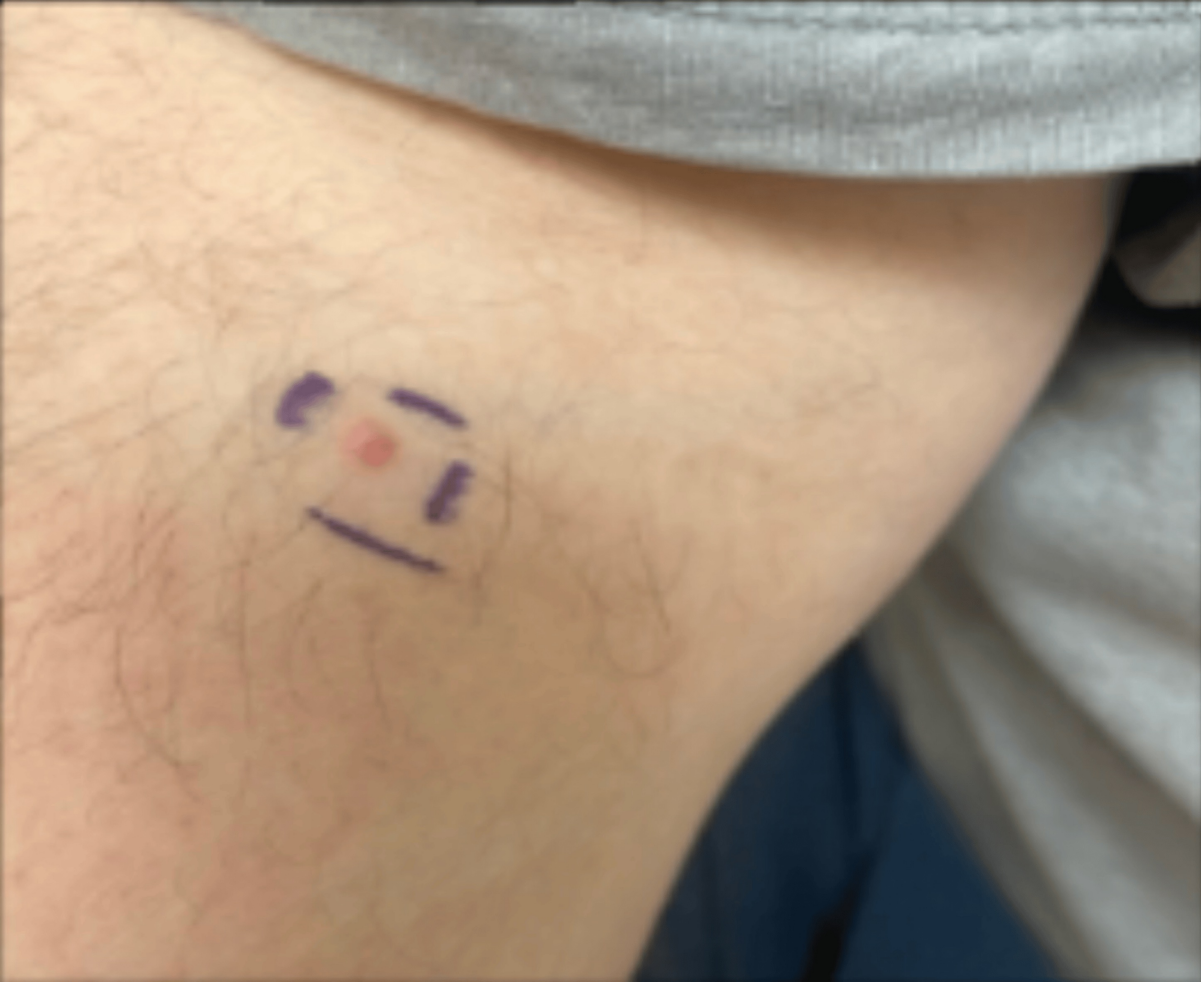
Red-yellow papule present on the upper extremity

We present a case of a Caucasian male patient with a past medical history significant for nonalcoholic steatohepatitis (NASH) and intestinal polyps who presented to the dermatology clinic with a red-yellow papule present on the upper extremity, depicted in Figure 1. The lesion was biopsied, and pathology revealed a diagnosis of sebaceous adenocarcinoma. The sample underwent immunohistochemistry (IHC) staining and was found to be negative for MLH1, MSH2, MSH6, and PMS2. The patient was treated with wide local excision and was referred to gastroenterology for further workup, including a scheduled fibrosure test, unrelated to his diagnosis of MTS. Urology consultation included urine cytology, which was negative for any evidence of hematuria or urothelial carcinoma. The patient has continuous follow-up with gastroenterology and urology services as a part of a multi-disciplinary approach.

## Discussion

MTS is a clinical diagnosis based on the presence of at least one sebaceous gland tumor (benign or malignant) and at least one Lynch syndrome-associated malignancy [2,4]. Additional diagnostics include histopathology consistent with sebaceous differentiation, germline genetic testing for MMR gene mutations, and microsatellite instability testing - the hallmark of MMR gene deficiency. IHC testing for MMR proteins remains a widely used screening method, though it does not distinguish somatic and germline mutations [2]. If both IHC and genetic testing are consistent with MTS, screening for internal malignancies is warranted [1]. IHC is used to detect loss-of-function mutations (LOF) in MMR genes, for instance, MLH1, MSH2, MSH6, and PMS2. LOF mutations result in the lack of MMR protein expression, which presents as absent staining on IHC, a delineating feature of MTS tumors [5]. It has also been suggested to perform germline testing for MMR gene defects as part of the initial workup of all immunocompromised patients who develop an MTS-associated neoplasm; especially if they have a current or past history of malignancy [4]. Individuals at risk for MTS may be identified using the Mayo MTS risk score, one of several diagnostic tools, as outlined in Table 1.

**Table 1:** Mayo Muir-Torre Scoring criteria for predicting Lynch syndrome Scoring interpretation: score ≥ 2 points: predicts an association with Lynch syndrome Reference: [5]

Criteria	Points
Age at presentation of initial sebaceous neoplasm	1 point if under 60 years old
Total number of sebaceous neoplasms or keratoacanthomas	2 points if two or more
Personal history of Lynch syndrome-associated internal malignancies	1 point
Personal history of Lynch syndrome-associated cancers	1 point

Management of MTS requires a multidisciplinary approach with interprofessional collaboration. Treatment of sebaceous tumors may involve local excision or cryotherapy while continuing to monitor for new lesions [1]. Sebaceous carcinomas can spread locally and to distant sites, necessitating more aggressive treatment with wide local excision or Mohs micrographic surgery [1]. Radiation therapy may be used as an adjunct after excision [1]. Additionally, combining interferon-alpha with oral isotretinoin has been shown to reduce the occurrence of cutaneous and visceral cancers [1]. Due to the high risk of internal malignancies, patients require regular screening and imaging studies tailored to the individual’s risk profile as outlined in Table 2 [1].

**Table 2:** Screening interventions or internal malignancies associated with MTS Reference: [2]

Screening recommendation	
Skin	Annually
Colonoscopy	Start at 20-25 years, and then every 1-2 years
Pelvic exams	Start at 30-35 years, and then annually
Upper endoscopy	Start at 30-35 years and then every 2-3 years
Urinalysis and cytologic examination	Start at 30-35 years, and then annually

Although MTS is a rare condition, there are other reports of similar presentations within the literature. Pancholi et al. reported a case of a 57-year-old female presenting with a 4 cm lesion on the left buttock. Histopathology revealed a sebaceous adenoma. The colonoscopy performed was significant for a malignant lesion at the ileocecal region, and biopsy results from the hemicolectomy were positive for a poorly differentiated signet ring adenocarcinoma with lymph node involvement [6]. Further, Shaker et al. reported a case of a 47-year-old woman who presented with multiple skin lesions involving the face, back, flanks, and scalp. Pathology results were significant for a sebaceous adenoma, and staining revealed a loss of MSH2 and MSH6. Continued workup revealed multiple tumors throughout the colon and rectum, with biopsy results significant for adenocarcinoma [2].

## Conclusions

Recognizing the cutaneous manifestations of MTS can lead to early identification and management of internal malignancies, as highlighted by this case. A multidisciplinary approach encompassing dermatology, gastroenterology, urology, and oncology is vital in early detection, management, and overall patient outcomes. Providers should maintain a high index of suspicion when encountering sebaceous tumors, especially in patients under 60 years old with a relevant personal or family history of Lynch syndrome-associated malignancies.
